# Heteroepitaxial growth of TiN film on MgO (100) by reactive magnetron sputtering

**DOI:** 10.1186/1556-276X-9-551

**Published:** 2014-10-03

**Authors:** Wei-Chun Chen, Chun-Yen Peng, Li Chang

**Affiliations:** 1 Instrument Technology Research Center, National Applied Research Laboratories, 20 R & D Road VI, Hsinchu Science Park, Hsinchu 30076, Taiwan; 2 Department of Materials Science and Engineering, National Chiao Tung University, Hsinchu, Taiwan

**Keywords:** Heteroepitaxial TiN, MgO (100), High quality, rf sputtering

## Abstract

TiN thin films were deposited on MgO (100) substrates at different substrate temperatures using rf sputtering with Ar/N_2_ ratio of about 10. At 700°C, the growth rate of TiN was approximately 0.05 μm/h. The structural and electrical properties of TiN thin films were characterized with x-ray diffraction (XRD), atomic force microscopy (AFM), scanning electron microscopy (SEM), transmission electron microscopy (TEM), and Hall measurements. For all deposition conditions, XRD results show that the TiN films can be in an epitaxy with MgO with cube-on-cube orientation relationship of (001)_TiN_ // (001)_MgO_ and [100]_TiN_ // [100]_MgO_. TEM with selected-area electron diffraction pattern verifies the epitaxial growth of the TiN films on MgO. SEM and AFM show that the surface of the TiN film is very smooth with roughness approximately 0.26 nm. The minimum resistivity of the films can be as low as 45 μΩ cm.

## Background

Titanium nitride (TiN) thin films have been extensively used in a wide range of applications as wear-protective coatings on mechanical components, cutting tools, decorations, as well as diffusion barriers and metal gates in integrated circuits [1,2], owing to its remarkable physical and chemical properties such as high hardness, high thermal stability, low electrical resistivity, and high wear excellent corrosion resistance [3,4]. Also, Pure TiN films are highly reflective and gold in color and have found applications in jewelery and optics [5]. Studies on TiN properties have solved significant problems, such as the reduction of the usefulness of TiN films for corrosion-resistant coatings and for diffusion barriers in the films [6]. Grigorov et al. reported that TiN has uses in microelectronics due to its efficiency in preventing aluminum diffusion into silicon in Al-TiN-Si trilayers at up to 550°C [7].

In order to deposit thin films of TiN on various substrates, it is common to use processes such as physical vapor deposition (PVD) [8], chemical vapor deposition (CVD) [9], atomic layer deposition (ALD) [10], and hallow cathode ionic plating (HCIP). Among those processes, the PVD process is known to be easy and to present a good adhesion between the film and the substrates of metals and ceramics [11]. Among various PVD processes, dc reactive sputtering is commonly used for deposition of polycrystalline and single-crystalline films. For epitaxial growth, TiN films have been often grown on an MgO substrate based on a consideration of the same rock salt structure and small lattice mismatch (<1% at room temperature) between their lattice parameters [12]. Previous studies of TiN deposition on the MgO (100) substrate showed that high-quality epitaxial TiN films can be obtained using ultrahigh vacuum dc reactive magnetron sputtering of pure Ti [13-16]. Reactive radio-frequency (rf) magnetron sputtering of a Ti target has been demonstrated for deposition of TiN polycrystalline films with preferred orientation dependence on argon/nitrogen ratio and substrate temperature [17]. In addition, Ingason et al. indicated that a minimum substrate temperature of 200°C is required for a good epitaxy. Also, substrate temperatures of 100°C and below yield low density, polycrystalline films with in-plane texture of ±12° around the main crystal axis of the MgO substrate [18].

In this article, we report heteroepitaxial growth of TiN deposited on MgO (100) using rf reactive magnetron sputtering in high vacuum at 600°C and 700°C. The crystallinity, surface morphologies, and microstructure of deposited films were characterized by x-ray diffraction (XRD), transmission electron microscopy (TEM), scanning electron microscopy (SEM), and atomic force microscopy (AFM).

## Methods

In our previous study of rf reactive sputtering of a Ti target for TiN polycrystalline films on Si (100), we found an optimum condition for growth of <100 > preferentially oriented TiN films [19] which was used for the present study for epitaxial growth of TiN films on MgO (100). TiN epilayers were deposited on the MgO (100) substrate using a 13.56 MHz rf sputtering apparatus without a bias voltage. MgO (100) of 2-in size was chosen as a substrate. The vacuum chamber was equipped with a cryopump to reduce the pressure to about approximately 1 × 10^-6^ Torr. An elemental Ti target (99.99% purity) was reactively sputtered in a mixture of Ar (99.999%) and pure N_2_ (99.9999%) with a ratio of 10:1, and the total gas pressure during the growth was 1 mTorr. The distance between the substrate holder and the target was 20 cm. The rf power was 200 W. MgO substrates were ultrasonically cleaned in acetone and ethanol and dried with dry N_2_ and immediately inserted into the vacuum chamber. Prior to TiN growth, the MgO (100) substrate was heat-treated in a vacuum of 5 × 10^-6^ torr at 700°C for 2 h, and the target was sputtered to clean the surface for 10 min with a shutter covering the substrate. Final, TiN epilayers were prepared on the MgO substrate at 600°C and 700°C. The epitaxial nature of the TiN (200) layer was examined using x-ray diffraction in ω-2*θ*, rocking curve (Siemens D5000, Siemens, Shinagawa-ku, Japan), and *ø*-scan mode with CuKα radiation (Bruker D8, Bruker Optik GmbH, Ettlingen, Germany). Surface morphology and microstrucure of deposited TiN in a cross section were analyzed in a field emission SEM (FE-SEM; Hitachi S-4300, Hitachi, Ltd, Chiyoda-ku, Japan) and TEM (Philips Tecnai 20, Philips, Amsterdam, The Netherlands). The cross-sectional TEM specimens were prepared by focused ion beam technique. The morphology of the film with surface roughness was investigated by AFM. Room-temperature electrical resistivities were determined with a four-point probe (Keithley 237, Keithley Instruments, Inc., Minato-ku, Japan).

## Results and discussion

**Figure 1:**
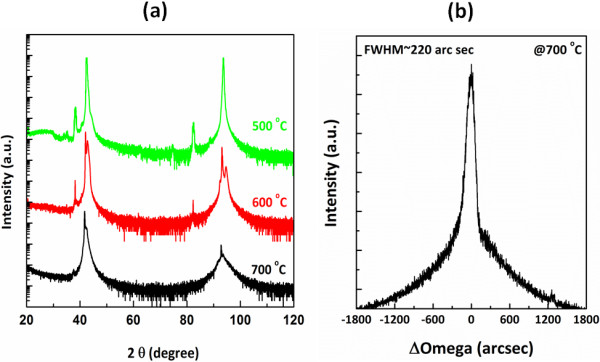
**XRD analysis of TiN films. (a)** XRD ω-2*θ* diffraction pattern and **(b)** (200) rocking curve of epi-TiN grown on MgO (100) at 700°C.

**Figure 2:**
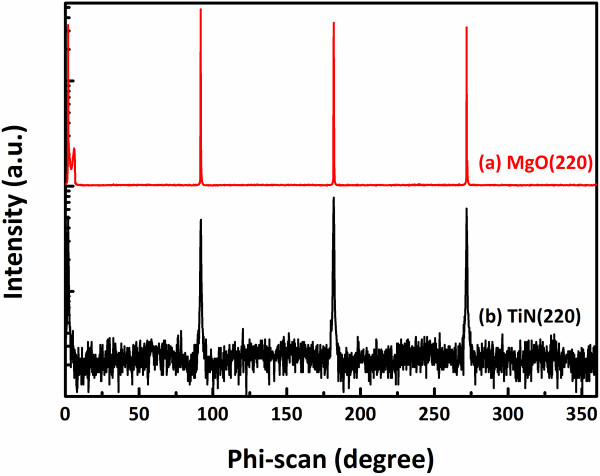
**Phi-scan patterns of XRD (220). (a)** MgO and **(b)** TiN film.

Figure 1a shows a ω-2*θ* x-ray diffraction pattern of TiN films grown on the MgO (100) substrate at 500°C to 700°C by reactive magnetron sputtering. It can be seen that the TiN peaks in the pattern exhibit only (200) and (400) reflections at 42.17° and 92.48° in the scanned 2*θ* range from 20° to 120°, suggesting the film is purely <100 > orientated in the direction normal to the substrate. The peaks of TiN and MgO (400) are separated in 0.9°, indicating the small lattice mismatch between them. While growth temperature is below 700°C, the TiN exhibit worse crystallinity. Specially, the TiN film grown on the MgO (100) substrate shows preferred orientation at 500°C. At 700°C, and from the peak positions of MgO, the lattice parameter of TiN in <100 > is deduced to be about 4.246 Å close to a stoichiometric value, implying that the strain of TiN film has been relaxed at the growth temperature [20]. The mismatch at room temperature between the TiN film and MgO substrate is estimated to be 0.8%. In order to better delineate the crystallinity of the TiN layer, an x-ray rocking curve was measured as shown in Figure 1b. The out-of-plane rocking curve of the TiN (200) peak shows the full width at half maximum (FWHM) of 220 arcsec, which is comparable to that of the MgO in 100 arcsec. Figure 2 shows the results of XRD phi-scans for the 220 peaks of MgO and TiN at a tilt angle of 45° with respect to the normal surface. Clearly, it is seen that both MgO and TiN have four 90°-distanced 220 peaks, showing the epitaxy of TiN on MgO with a cube-on-cube relationship of TiN (100) // MgO (100) and TiN [100] // MgO [100]. The results suggest that the TiN film on MgO (100) can be epitaxially grown with high quality, in comparison with our previous studies of TiN deposition on Si (100) studies in the same deposition condition [19].

**Figure 3:**
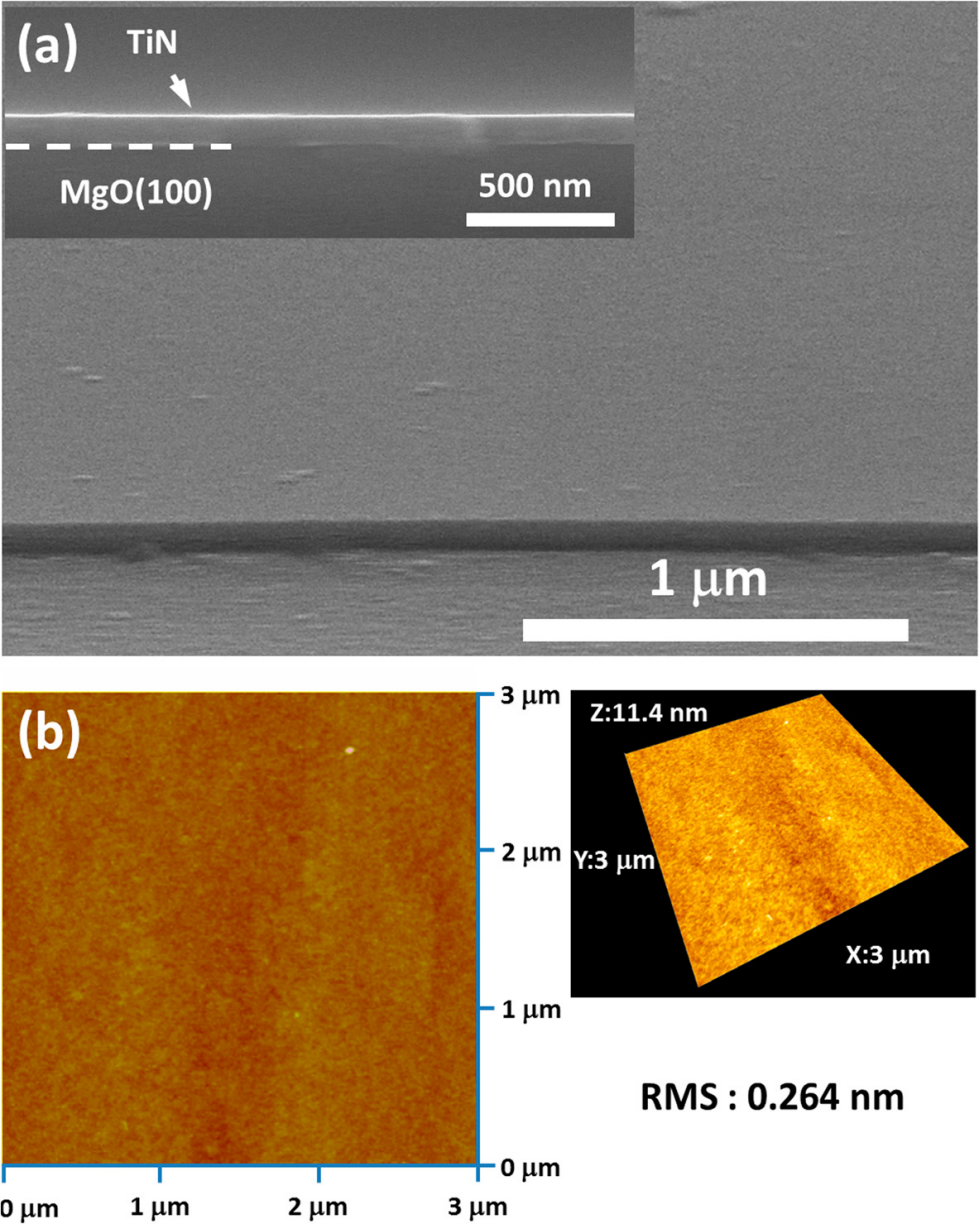
**Surface morphology of the TiN film. (a)** SEM in a tilted view with inset in a cross section and **(b)** AFM images.

The surface morphology of the TiN film is shown in SEM and AFM images (Figure 3). The SEM image in Figure 3a from a tilted view shows that the TiN surface is smooth, and the cross-sectional SEM in the inset illustrates that the uniform thickness of the TiN thin film on the MgO substrate is about 100 nm, giving the growth rate of 50 nm/h. Also, the surface roughness of TiN measured by AFM in Figure 3b of 3 × 3 μm^2^ area is about 0.26 nm in a root-mean-square value slightly greater than that of MgO in 0.13 nm after 700°C heat treatment in vacuum.

**Figure 4:**
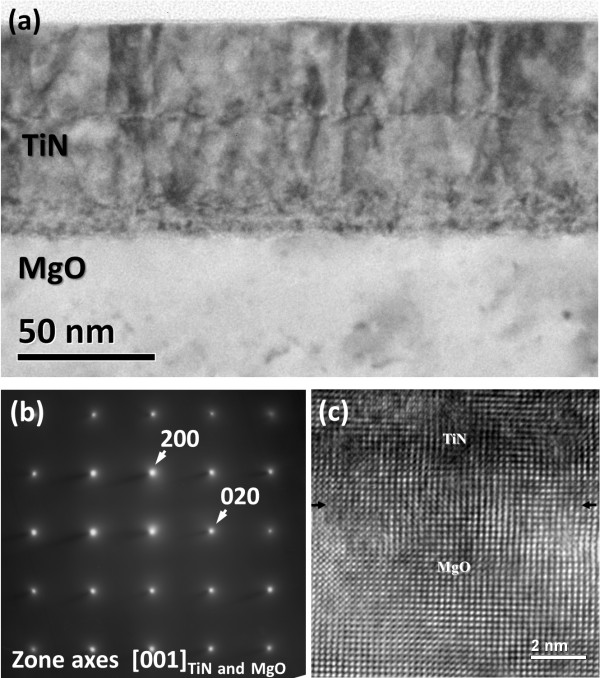
**TEM images of the cross section of the TiN/MgO. (a)** Cross-sectional bright-field TEM micrograph. The discontinuous features at the middle region of TiN and at the TiN/NgO interface were artifacts due to FIB milling. **(b)** a corresponding SAED pattern from an epitaxial TiN film grown on the MgO (100) substrate. **(c)** High-resolution TEM showing the TiN/MgO interfacial region.

Figure 4 shows a cross-sectional bright-field TEM image of the TiN/MgO (100) interface. The image also shows the smoothness of the TiN film at the surface and the interface with MgO. The dislocation density is roughly estimated in the order of magnitude of 10^11^ cm^-2^. Between MgO and TiN, a sharp interface can be seen. It should be noted that the layer-like image contrast around the interface region is due to artifacts from FIB milling in a number of steps as HRTEM and diffraction data show no evidence for the existence of other phases. The epitaxial growth of the TiN film on the MgO substrate is further confirmed with the result of the corresponding selected-area diffraction (SAD) pattern from the interfacial region as shown in Figure 4b. The SAD pattern illustrates that both of {002}-type spots of TiN and MgO are nearly coincidental due to the small lattice mismatch, while some of {004} spots are slightly split in a detailed examination. Also, no diffraction spots from extra phases have been observed, suggesting that no interlayer reaction occurs between TiN and MgO. Further, the coherency of TiN with MgO can be observed in Figure 4c of an HRTEM image from the interfacial region. As the misfit dislocations can be separated in about 26 nm, the image reveals most of the good coherent regions along the interface. Also, it is noticed that more defects appear at the region above 2-nm thickness from the interface, suggesting that the crystallinity may decrease with film growth. Further improvement may require varying the Ar/N ratio and the deposition rate.

Resistivity measurements can be used to qualitatively determine the defect concentration in a metal film. Also, the resistivity of TiN films depends on microstructure, preferred orientation, and stoichiometry. TiN films exhibit resistivity of 45 μΩ cm in average at room temperature higher than the bulk crystal value of 13 μΩ cm. The result is in agreement with the reports of Yokota et al. [21] and Biunno et al. [22]. In contrast, polycrystalline TiN films deposited on Si (100) in the same sputtering condition have the resistivities in the range of 200 μΩ cm, implying that grain boundaries have strong effects on electron scattering.

Compared to other studies on electrical resistivity of TiN, the value is very close to present accepted values. Therefore, the result implies that the film quality is reasonably good.

## Conclusions

In summary, high-quality epitaxial TiN (100) films can be deposited on a 2-in MgO (100) substrate at 700°C by rf reactive sputtering process in a high vacuum. Structural characterization shows that epitaxial TiN films exhibit a smooth surface and a coherent good interface with MgO. Also, TiN films of 100-nm thickness are close to stoichiometry and strain-relaxed.

## Competing interests

The authors declare that they have no competing interests.

## Authors’ contributions

WCC designed and carried out the experiment, statistical analysis, and participated in the draft of the manuscript. CYP carried out the high-resolution X-ray measurements. LC was involved in the discussions of experimental results. All authors read and approved the final manuscript.
